# Clinical Relevance of PCR Versus Culture in Urinary Tract Infections Diagnosis: Quantification Cycle as a Predictor of Bacterial Load

**DOI:** 10.3390/diagnostics15151939

**Published:** 2025-08-01

**Authors:** Pallavi Upadhyay, Arjuna Vallabhaneni, Edward Ager, Barbara Alexander, Adriana Rosato, Vijay Singh

**Affiliations:** 1 HealthTrackRx, 1500 I-35 W, Denton, TX 76207, USA; 2 Division of Infectious Diseases, Departments of Medicine and Pathology, Duke University, Durham, NC 27701, USA; 3 Department of Medicine, Maine Health Institute for Research, Scarborough, ME 04074, USA

**Keywords:** urinary tract infections, multiplex PCR syndromic panel testing, urine culture, quantification cycle, colony forming units

## Abstract

**Background**: Unambiguous clinical interpretation of PCR results for urinary tract infections (UTIs) remains a challenge. Here we compare and correlate multiplex qPCR results (quantification cycle values) with traditional microbial culture results (colony forming units) for clinical samples. **Methods**: Serial dilutions [10^8^ to 10^0^ colony forming units (CFU)/mL] were performed on five Gram-negative and two Gram-positive UTI-causing bacterial pathogens. For each dilution, quantitative cultures on solid media to confirm CFU/mL values and a real-time PCR UTI panel employing a nanofluidic Open Array^TM^ platform producing quantification cycle (Cq) values were performed. Cq values were correlated with CFU/mL values, generating a semi-quantitative interpretive scale for clinical samples. The clinical utility of the scale was then assessed using PCR and culture data from 168 clinical urine samples. **Results**: For Gram-negative bacteria, Cq values of <23, 23 to 28, and >28 corresponded with ≥10^5^ CFU/mL, <10^5^ CFU/mL and negative cultures, respectively. For Gram-positive bacteria, Cq values of <26, 26 to 30, and >30 corresponded with ≥10^5^ CFU/mL, <10^5^ CFU/mL and negative cultures, respectively. Among 168 urine specimens (including 138 Gram-negative and 30 Gram-positive bacteria), there was 83.3% agreement (*n* = 140/168) and 16.6% non-agreement (*n* = 28/168) between culture CFU/mL and qPCR Cq. Gram-negative bacteria had higher agreement (87.6%, 121/138) than Gram-positive bacteria (63.3%, 19/30). **Conclusions**: This study demonstrates that qPCR Cq results can be directly correlated with traditional urine quantitative culture results and reliably identify the clinically relevant cutoff of 10^5^ CFU/mL for detected uropathogens.

## 1. Introduction

Urinary tract infections (UTIs) have been routinely diagnosed by standard urine culture (SUC), which comes with inherent limitations as a diagnostic test. While SUC is considered the “gold standard” for UTI testing, cultures take time for incubation, identification and susceptibility testing of organisms recovered. Furthermore, it is reported that accuracy of SUC in identifying UTIs in symptomatic patients is approximately 60% [1]. Recently, advanced clinical diagnostic technologies, including expanded quantitative urine cultures (EQUC), next generation sequencing (NGS) and nucleic acid amplification tests (NAAT) such as quantitative polymerase chain reaction (qPCR) and real time qPCR, have been developed to address the low sensitivity and prolonged turnaround time of results associated with SUCs [2].

While each of these advanced technologies help address specific challenges of SUC, each also has unique impediments to routine clinical use. For example, EQUC methods enable detection of a broader range of viable bacteria and fungi, but do not improve testing turnaround time [3,4,5]. NGS-based testing provides greater sensitivity and accuracy; however, it is not widely used in routine UTI diagnosis [5], as the requirement of complex data analysis and specialized expertise for testing renders NGS-based UTI testing both time and cost-intensive [6]. qPCR-based molecular testing can overcome several of the challenges that SUC presents, including the ability to detect anaerobic, intracellular, and non-bacterial infections, accurate identification of all causative organisms (even those in low concentrations) in a clinical specimen, and with significantly faster turn-around time (often same-day results). Furthermore, a recent meta-analysis reported that qPCR had higher specificity and sensitivity for UTI pathogen detection compared with NGS [7]. However, despite the potential advantages of qPCR, one of the biggest challenges facing adoption of qPCR testing for UTI diagnosis in the clinical setting is its enhanced sensitivity, particularly when applied to a clinical specimen for which contamination with potential uropathogens is not uncommon. In this setting, quantifying the amount of organism recovered in an SUC has historically helped guide interpretation and treatment decisions.

With SUC, a quantitative threshold of ≥10^5^ CFU/mL of bacteria is regarded as clinically significant in freshly voided urine. Bacterial counts between 10^4^ and 10^5^ CFU/mL are generally interpreted according to clinical status. Bacterial counts < 10^4^ CFU/mL are considered to have lower probability of UTI [8]. Recent studies indicate that bacterial counts as low as 10^2^ CFU/mL can cause UTI in symptomatic patients [9,10,11]. Thus, understanding how qPCR results correlate with that of traditional quantitative SUC results would be beneficial in helping to interpret the qPCR result in the context of the patient’s clinical presentations and symptoms, and ultimately, in understanding which pathogens may or may not require treatment.

To address this challenge, we performed real-time qPCR testing on previously cultured urine samples and compared (colony forming unit per milliliters [CFU/mL] versus quantification cycle [Cq]) for each sample to aid in PCR result interpretation and to enhance clinical applicability.

## 2. Materials and Methods

### 2.1. Bacterial Strains

Bacterial strains (Table 1) were obtained from ThermoFisher (Lenexa, KS, USA) and were cultured on Tryptic Soy Agar (TSA) (Cat# R01624) or Mueller-Hinton Agar (MHA) (Cat# R111816) purchased from Remel (Lenexa, KS, USA).

### 2.2. Quantification of Bacterial Load in Urine Samples Using Culture-Based Methods

Contrived urine samples with various bacterial concentrations were prepared as follows. Using bacterial colonies grown overnight on TSA plates, an initial cell suspension equivalent to 0.5 McFarland (1.5 × 10^8^ CFU/mL) was prepared in sterile urine by adjusting the cell density using a densitometer. A series of seven tenfold dilutions (1.5 × 10^7^ to 1.5 × 10^0^) were prepared in sterile urine. For each dilution, 100 µL was inoculated on either TSA or MHA in triplicate and incubated overnight at 37 °C. Colony counts were performed when discernible colonies were present; confluent growth was recorded as “too numerous to count” (TNTC). The experiment was repeated three times to ensure reproducibility.

### 2.3. qPCR to Determine the Cq Value for Known Bacterial Population Estimate

For each serial dilution, 1.0 mL was aliquoted in a 1.5 mL micro-centrifuge tube and centrifuged at 10,000× *g* for 10 min. The supernatant was decanted, and the cell pellet resuspended in 200 µL of PrimeStore^TM^ molecular transport medium (Longhorn Diagnostics, MD, USA). Total nucleic acid was extracted into 50 µL elution buffer using ThermoFisher KingFisher™ Flex (ThermoFisher Scientific, San Jose, CA, USA) system following the manufacturer’s protocol without any modification. Using 8 µL of extracted nucleic acid, qPCR was carried out using the HealthTrackRx OpenArray™ UTI syndromic panel (Denton, TX, USA) (Table 2) in triplicate for each dilution using the protocol described previously [12]. The OpenArray™ cards were run on ThermoFisher QuantStudio™ 12K Flex platform (ThermoFisher Scientific, CA, USA) with the following PCR cycling conditions: initial Enzyme activation at 95 °C for 10 min followed by 40 cycles of denaturation at 95 °C for 15 s followed by annealing/extension at 60 °C. The OpenArray™ technology is a high-throughput nanofluidic platform that uses custom arrays pre-plated with TaqMan^TM^ assays for the detection of target pathogens via PCR. The performance of PCR assays with respect to limit of detection, sensitivity and specificity were performed according to well-established Clinical and Laboratory Standards Institute (CLSI) guidelines for multiplex PCR assays.

### 2.4. Establishing the Cq to CFU/mL Correlation Algorithm

Using results from the contrived urine specimens, and considering culture quantification as the standard, an algorithm was developed to correlate CFU/mL with Cq values as follows: the serially diluted bacteria were plated in triplicate at each dilution. Bacterial colonies were counted on each culture plate and counts were recorded. The average across three plates for each dilution was recorded and multiplied by a factor of ten to give CFU/mL. qPCR was performed in triplicate for each dilution to generate Cq values. The average of the three Cq values for each dilution was calculated. Standard curves for each pathogen target were established by plotting the Cq values against the CFU/mL data for each dilution. A correlational algorithm was then established to define a clinically relevant threshold of 10^5^ CFU/mL based on Cq values obtained through qPCR according to the linear equation obtained through the standard curves.

### 2.5. Correlation of Cq and CFU in Clinical Specimens

Residual and de-identified clinical urine samples were obtained from ARUP Labs, Utah, USA. Samples were collected over a period of two months in a blind fashion to avoid any bias. No patient information was collected and only the concordance between the microbial culture and PCR results was calculated based on the detection of the pathogens. A study comparing the qualitative results of urine culture and qPCR for the specimen cohort was previously published [13]. In the current study, we retrospectively analyzed the concordance between Cq and CFU/mL in the 168 specimens for which both traditional urine culture data and Cq values using UTI syndromic qPCR were available. Correlation was performed for all bacterial isolates based on their Gram staining properties using the developed algorithm.

### 2.6. Statistical Analysis

Categorical variables are presented as numerical values (*n*, %). Error bars wherever presented depict the standard deviation. Statistical analyses were performed using R version 4.3.2 (R Foundation for Statistical Analysis, Vienna, Austria).

## 3. Results

### 3.1. Estimation of Colony-Forming Units from Serially Diluted Bacterial Cell Suspensions

**Figure 1:**
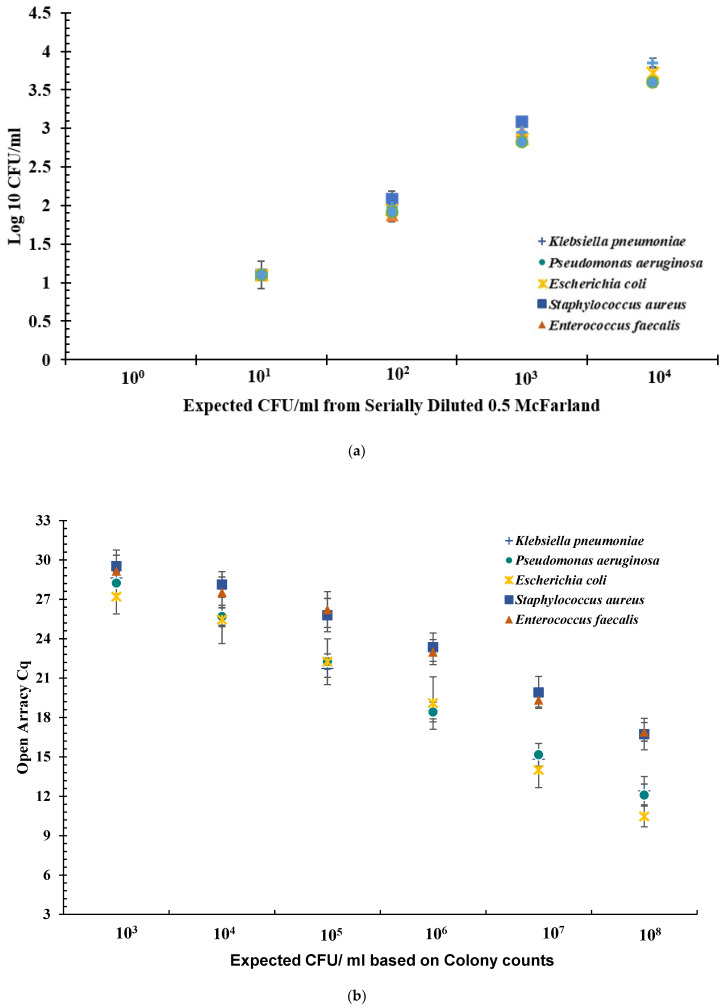
(**a**) **Log10 CFU/mL for serially diluted bacterial cell suspension.** The serially diluted bacteria were plated in triplicate at each dilution. All counts were recorded. The Average across three plates was recorded and multiplied by factor of ten to give CFU/mL. The value CFU/mL was transformed into Log10 value and plotted. The error bars indicate the standard deviation across three experiments of Log10 value of CFU/mL. Error bars represent standard deviation. (**b**) Open Array^TM^ quantification cycle (Cq) values obtained at dilutions 10^3^ to 10^8^ CFU/mL for five bacteria spiked in urine. Sterile urine spiked with bacterial cells with concentrations ranging from 10^8^ CFU/mL to 10^0^ CFU/mL assayed for Cq values using qPCR. Each experiment was performed in triplicate. The data shown here is an average of three independent experiments. Error bars indicate standard deviation across three independent experiments. Error bars represent standard deviation.

To quantify bacterial load across serial dilutions, CFU/mL values were log-transformed (Log_10_) and plotted against the expected cell density based on turbidity measurements (Figure 1a). The dilution series began with a 0.5 McFarland standard, corresponding to 1.5 × 10^8^ CFU/mL.

Optical density measurements provided additional insight into bacterial concentration. When the expected Log_10_ (CFU/mL) value was 3.17, the average optical density for Gram-negative bacteria was 2.87 (±0.05), while for Gram-positive bacteria it was 3.02 (±0.03). At culture densities exceeding 10^5^ CFU/mL, colony growth became too confluent for accurate enumeration, and these values were recorded as TNTC and excluded from further analysis.

### 3.2. Determination Cq Values by Open Array™ Multiplex qPCR

At bacterial cell densities of 10^4^ and 10^5^ CFU/mL, the following Cq values were observed: *E. faecalis* 27.5 (±1.9) and 26.2 (±2.4), *S. aureus* 28.1 (±1.3) and 25.7 (±2.4), *E. coli* 25.4 (±2.7) and 22.2 (±2.7), *K. pneumoniae* 25.7 (±1.4) and 21.7 (±1.4), *P. aeruginosa* 25.7 (±1.0) and 22.2 (±0.9), respectively. On average, Gram-positive bacteria showed distinctively different Cq values to that of the Gram-negative bacteria tested. In the case of Gram-negative bacteria at 10^5^ CFU/mL, the average Cq value was 21.8 (±1.7), whereas for Gram-positive bacteria it was 26.5 (±2.4). Similarly, at 10^4^ CFU/mL for Gram-negative bacteria, the average Cq was 25.6 (±1.6), whereas for Gram-positive bacteria it was 27.9 (±1.5) (Figure 1b).

### 3.3. Cq to CFU/mL Correlation Algorithm

Based on the data from Cq to CFU/mL experiments (Figure 1a,b), a clinically relevant cutoff of 10^5^ CFU/mL was determined on the basis of Cq values generated via qPCR. Per Table 3, for Gram-negative bacteria, Cq values of <23 corresponded with ≥10^5^ CFU/mL and Cq values between 23 and 28 corresponded with <10^5^ CFU/mL. For Gram-positive bacteria, Cq values of <26 corresponded to ≥10^5^ CFU/mL and Cq values between 26 and 30, corresponded with <10^5^ CFU/mL. With respect to the urine culture standards, Cq > 28 for Gram-negative bacteria and Cq > 30 for Gram-positive bacteria corresponded to negative.

We then retrospectively applied the Cq to CFU/mL criteria established using the three Gram-negative (*E. coli, K. pneumoniae* and *P. aeruginosa*) and two Gram-positive bacteria (*S. aureus* and *E. faecalis*) (Table 3) to the 168 residual clinical samples. Among 138 urine samples that grew Gram-negative pathogens, 116 resulted as ≥10^5^ CFU/mL and among these, 89.6% (*n* = 104) displayed a Cq value of <23, which directly correlated to 10^5^ CFU/mL range. In addition, of the remaining 12 Gram-negative bacteria that resulted as 10^5^ CFU/mL, six had Cq values greater than 23 were all within the ±2 standard deviation (SD) observed in our study. Of 30 urine specimens that grew Gram-positive bacteria, 15 were quantified as greater than 10^5^ CFU/mL and among those, 11 were below the PCR Cq value of 26. Overall, there was 83.3% agreement (*n* = 140/168) and 16.6% non-agreement (*n* = 28/168) between culture CFU/mL and Cq to CFU/mL. For Gram-negative isolates (*n* = 138), there was 87.6% (121/138) agreement and 12.3% (17/138) disagreement. Whereas, for Gram-positive bacteria (*n* = 30), the agreement was 63.3% (*n* = 19/30) and disagreement was 36.6% (*n* = 11/30) (Table 4).

**Figure 2:**
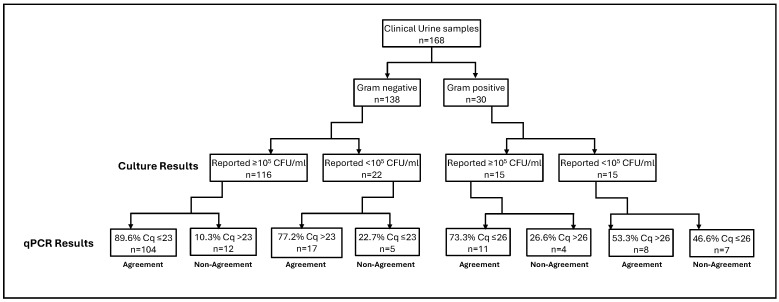
**Cq-CFU/mL clinical validation.** Cq values obtained from qPCR tests were compared to the previously tested urine samples via microbial culture. A Cq cutoff of ≤23 (for Gram-negative) and ≤26 (for Gram-positive) was used to establish correlation with culture results above or below 10^5^ CFU/mL. The figure summarizes agreement/non-agreement between qPCR and microbial culture techniques reported in Table 4.

The average Cq value detected for the five bacteria species used to develop the correlation algorithm all agreed with the proposed threshold values (Table 3). For *E. coli* the average Cq value detected in cultures reported as >10^5^ CFU/mL was 17.99 (range 12.97 to 27.66). For *Klebsiella* the average value was 20.82 (range 17.11 to 23.89). The three *Pseudomonas* isolates gave an average Cq value of 22.61 (range 21.64 to 26.469). Two *Enterococcus* species detected had an average Cq value of 22.47 (19.94 and 25) and *S. aureus* Cq values averaged 21.26 (range 14.73 to 28.19). In cultures reported as <10^5^ CFU/mL, thirteen *E. coli* were detected with an average Cq of 25.26 (range 18.69 to 28.46). The average for *Klebsiella* Cq values was 27.4 (range 23.51 to 19.25), and *Enterococcus*, 27 (26 to 29.7). The Cq-CFU/mL threshold algorithm developed as part of this study was also applied on Gram-negative and Gram-positive bacterial pathogens which were not part of the Cq-CFU/mL correlation but that were detected by the multiplex PCR panel. A total of twenty-one isolates, sixteen Gram-negative and five Gram-positive bacteria were analyzed. Thirteen isolates showed positive agreement between reported CFU/mL values and CFU/mL values derived using the threshold values. All but one *Proteus* isolate (1/13) showed positive agreement for specimens reported as greater than 10^5^ CFU/mL. Four *S. agalactiae* (GBS), one *Enterobacter* and two *Citrobacter* isolates were reported with CFU/mL values less than 10^5^ CFU/mL which, following application of our proposed threshold values, the Cq values detected would have correlated to a CFU/mL value greater than 10^5^ CFU/mL (Table 4, Figure 2).

## 4. Discussion

To improve the clinical efficacy of multiplex qPCR-based UTI detection and to provide a tool for clinicians to reliably interpret UTI PCR results in relation to traditional culture results, this study aimed to correlate the multiplex UTI PCR values (Cq) with colony counts (CFU/mL) of urine cultures. Unlike molecular panels for other infectious syndromes, fewer studies have compared multiplex PCR to traditional urine culture for diagnosing UTIs [14,15,16]. For comparison, 100,000 CFU/mL (10^5^) was set as the target comparator as this value is widely considered a clinical diagnostic cutoff for diagnosing UTIs in symptomatic patients [5].

The results from our study showed a CFU/mL value of ≥10^5^ correlated with average Cq values below 22.5 for all Gram-negative bacteria assessed, whereas for Gram-positive bacteria the Cq value that best correlated to ≥10^5^ CFU/mL was 26.5 (Table 3). Our results are in agreement with previously published studies wherein multiplex PCR-based detection of UTI-causing pathogens was directly compared to those detected via culture in a clinical setting [17]. In a similar comparative analysis of Cq and CFU/mL values for bacterial pathogens in bronchial samples, Burillo and co-workers reported 24.7 as the Cq value that best corresponded to 10^5^ CFU/mL. The authors also described 10^4^ CFU/mL as corresponding to an average Cq value of 26.9 for Gram-negative bacteria [18].

The derived semi-quantitative thresholds correlating Cq values to urine culture CFU/mL values were validated using a collection of clinical specimens previously submitted for urine culture and reported [13]. Based on the results of our Cq-CFU/mL study (Table 3), we used a Cq value of 23 for Gram-negative bacteria and Cq of 26 for Gram-positive bacteria as the semi-quantitative criteria to approximate a CFU/mL of ≥10^5^. After converting Cq values to CFU/mL for previously tested urine isolates, we looked at the agreement between the two sets of CFU/mL values. The percent agreement between the results obtained after applying the threshold values was 83.3% (140/168) for the five bacterial strains used in this study (Table 4). Despite the overall high agreement, we observed 36.6% disagreement for Gram-positive bacteria. This can be attributed to differences in overall PCR efficiency, assay design and the small number of Gram-positive strains utilized to derive the Cq value scale which underscores the need for further studies with additional bacterial strains and qPCR assays to establish a more robust interpretation scale.

A major concern with PCR-based testing is the detection of pathogens in samples that are deemed culture negative [14,15,16], suggesting that PCR may be too sensitive a technique for reliably reporting clinically relevant levels of uropathogens. In general, the decision-making process of appropriate diagnostic tests for UTIs (based on clinical symptoms) as well as result interpretation for timely treatment remains a challenge [19]. Furthermore, the high sensitivity of molecular tests in comparison to traditional culture techniques complicates clinical interpretation of the results and remains a significant barrier in the widespread adoption of PCR for the detection of UTIs. Since PCR can detect nucleic acid material from non-viable and degraded pathogens, a positive PCR result may not reflect a true UTI even in symptomatic patients [20]. Similarly, in cases of a culture-negative but PCR-positive diagnosis, a clear distinction between infection and colonization cannot be made without further research into the clinical validity of the quantitative PCR test [17]. A study comprising 220 women with UTI symptoms demonstrated that 95.9% samples were positive for *E. coli* via PCR versus 80.9% cultures positive for any UTI-causing pathogen [17]. In our study, among the previously tested urine culture specimens, those reported as negative by culture were also analyzed. The multiplex UTI PCR panel included pathogens not readily detected in traditional culture, and several were observed in the study. Notably, there were 59 culture-negative specimens where *E. coli* was detected via PCR [13]. Upon applying our proposed threshold values for Cq-CFU/mL, 93.2% (*n* = 55/59) of these PCR-positive *E. coli* samples were interpreted to be less than 10^5^ CFU/mL and only four had Cq values that would have been reported (≥10^5^ CFU/mL). These findings suggest that our proposed semi-quantitative criteria, when applied to Cq values from patient specimens, reliably correlates to CFU/mL cutoff values used in the clinical diagnosis of UTIs and may help delineate pathogens from non-pathogenic and/or non-viable colonizers.

Previous studies have argued that PCR is a reliable and beneficial replacement for traditional urine culture [17,18,21]. Our study shows that a robust correlation between Cq and CFU/mL results exists, and the correlation can be effectively incorporated into result reports to assist in patient diagnosis and to enable more targeted approaches to antimicrobial therapy.

The implementation of syndromic PCR panels has had a significant impact on rapid disease diagnosis [22]. In support of NAATs, it has been opined that large multiplex PCR panels should be the first-line tests for detection of respiratory and intestinal pathogens [23]. A single-center, 150-patient study showed the standard urine culture (at 10^5^ CFU/mL) displayed poor sensitivity and missed 67% of all detected UTI-causing pathogens, indicating a need for more sensitive methods for pathogen detection in urine specimens [3]. The increasing demand for high-throughput tests that can detect a range of diverse pathogens has further placed multiplex testing at the forefront of options sought by the modern clinical laboratory [2,24]. This testifies to the growing importance of multiplex PCR tests as an effective tool for uropathogen detection in clinical samples. Despite the increased sensitivity and specificity, a major disadvantage of PCR is its limitation of detecting only known resistance genes and the technology in its current format cannot be used for assessing true antibiotic sensitivity of a detected pathogen as opposed to established phenotypic susceptibility testing methods. However, when interpreted in conjunction with the clinical presentation and examination, qPCR tests may streamline testing, optimize antibiotic use and potentially improve patient outcomes [25,26].

We recognize that the current study has limitations. Firstly, the number of pathogens used to validate our Cq-CFU/mL threshold algorithm is limited. Further studies using more bacterial strains, particularly Gram-positive species, are currently being performed to support the observations made in this study. Secondly, the clinical validation on clinical samples was performed on residual samples. Another limitation of our study is the non-standardized nature of the PCR assay used, and the results cannot be universally utilized without incorporating multi-site testing. To further demonstrate the true clinical effectiveness of our correlation algorithm, prospective studies with defined patient populations are needed wherein urine culture is compared to the Open Array^TM^ multiplex PCR panel simultaneously which would enhance the observations made in this study.

## 5. Conclusions

Molecular testing for UTIs, when compared to traditional culture techniques, presents advantages with respect to increased sensitivity and rapid turnaround time for results. However, widespread acceptance of this methodology is hampered by limitations of PCR-based results for establishing clinically relevant cutoffs of the detected uropathogens. Our results establish a clear agreement between the PCR-generated Cq value and the traditional CFU/mL value generated via microbial culture. Validation of our semi-quantitative interpretive scale using clinical samples demonstrates that PCR Cq values correspond with established standard of care quantitative culture thresholds, providing physicians with valuable additional information to aid in interpreting qPCR results for the diagnosis of UTIs.

## Figures and Tables

**Table 1:** Five common UTI-causing bacteria used in this study.

Bacteria	Strain	Source
*Escherichia coli*	ATCC 25922	ThermoFisher (KS, USA)
*Klebsiella pneumoniae*	ATCC 700603	
*Pseudomonas aeruginosa*	ATCC 27853	
*Staphylococcus aureus*	BA 977	
*Enterococcus faecalis*	ATCC 51299	

**Table 2:** Pathogens included on the custom Open Array^TM^ Multiplex PCR UTI panel.

Bacterial Pathogens	
*Acinetobacter baumannii*	*Staphylococcus aureus*
*Citrobacter freundii*	*Staphylococcus epidermidis*
*Enterobacter cloacae complex*	*Staphylococcus haemolyticus*
*Enterobacter aerogenes*	*Staphylococcus lugdunensis*
*Enterococcus faecalis*	*Staphylococcus saprophyticus*
*Enterococcus faecium*	*Streptococcus agalactiae* (Group B Strep)
*Escherichia coli*	*Streptococcus pyogenes* (Group A Strep)
*Klebsiella pneumoniae*	
*Klebsiella oxytoca*	
*Morganella morganii*	**Fungal Pathogens**
*Proteus mirabilis*	*Candida albicans*
*Proteus vulgaris*	*Candida parapsilosis*
*Pseudomonas aeruginosa*	*Candida glabrata*
*Serratia marcescens*	*Candida krusei*

**Table 3:** Proposed reporting criteria for Cq to CFU/mL on clinical samples.

Bacterial Concentration	Cq Value Range	Report
Gram-Negative Bacteria		
≥10^5^ CFU/mL	<23	Detected ≥ 10^5^ CFU/mL
<10^5^ CFU/mL	23–28	Detected < 10^5^ CFU/mL
NA	>28	Negative
**Gram-Positive Bacteria**		
≥10^5^ CFU/mL	<26	Detected ≥ 10^5^ CFU/mL
<10^5^ CFU/mL	26–30	Detected < 10^5^ CFU/mL
NA	>30	Negative

**Table 4:** Agreement/non-agreement between culture-derived CFU/mL and Cq to CFU/mL correlation.

	Culture (CFU/mL) Result	Count	Agreement	Non-Agreement	Percentage
***E. coli***	≥10^5^	90	82	8	91.1
	<10^5^	13	11	2	84.6
***Klebsiella* spp.**	≥10^5^	11	9	2	81.8
	<10^5^	5	5	0	100
***Pseudomonas* spp.**	≥10^5^	3	2	1	66
	<10^5^	0	0	0	NA
***Enterobacter* spp.**	≥10^5^	6	6	0	100
	<10^5^	1	0	1	0
***Proteus* spp.**	≥10^5^	4	3	1	75
	<10^5^	1	1	0	100
***Serratia marcescens***	≥10^5^	1	1	0	100
	<10^5^	0	0	0	N/A
***Citrobacter* spp.**	≥10^5^	1	1	0	100
	<10^5^	2	0	2	0
***Staphylococcus aureus***	≥10^5^	4	3	1	75
	<10^5^	0	0	0	NA
***Enterococcus* spp.**	≥10^5^	4	4	0	100
	<10^5^	5	4	1	80
***Streptococcus agalactiae***	≥10^5^	1	1	0	100
	<10^5^	4	0	4	0
***Coagulase Negative Staphylococcus* spp.**	≥10^5^	6	3	3	50
	<10^5^	6	4	2	67
	**Total**	**168**	**140**	**28**	

## Data Availability

The original contributions presented in the study are included in the article and further inquiries can be directed to the corresponding authors.

## Abbreviations

Cq	Quantification cycle
CFU	Colony forming unit
EQUC	Expanded quantitative urine cultures
MHA	Mueller-Hinton agar
NAAT	Nucleic acid amplification tests
NGS	Next generation sequencing
qPCR	Quantitative PCR
SUC	Standard urine culture
TNTC	Too numerous to count
TSA	Tryptic soy agar
UTI	Urinary tract infection
